# Prediction of vulnerability to bipolar disorder using multivariate neurocognitive patterns: a pilot study

**DOI:** 10.1186/s40345-017-0101-9

**Published:** 2017-09-01

**Authors:** Mon-Ju Wu, Benson Mwangi, Ives Cavalcante Passos, Isabelle E. Bauer, Bo Cao, Thomas W. Frazier, Giovana B. Zunta-Soares, Jair C. Soares

**Affiliations:** 1 UT Center of Excellence on Mood Disorder, Department of Psychiatry and Behavioral Sciences, The University of Texas Science Center at Houston, Houston, TX USA; 2 Cleveland Clinic Children’s Hospital Center for Pediatric Behavioral Health, Cleveland, OH USA; 3 Department of Psychiatry & Behavioral Sciences, The University of Texas Health Science Center, 1941 East Road, Houston, TX 77054 USA

**Keywords:** Bipolar disorder, Neurocognition, Vulnerability, CANTAB, Machine learning

## Abstract

Bipolar disorder (BD) is a common disorder with high reoccurrence rate in general population. It is critical to have objective biomarkers to identify BD patients at an individual level. Neurocognitive signatures including affective Go/No-go task and Cambridge Gambling task showed the potential to distinguish BD patients from health controls as well as identify individual siblings of BD patients. Moreover, these neurocognitive signatures showed the ability to be replicated at two independent cohorts which indicates the possibility for generalization. Future studies will examine the possibility of combining neurocognitive data with other biological data to develop more accurate signatures.

**Electronic supplementary material:**

The online version of this article (doi:10.1186/s40345-017-0101-9) contains supplementary material, which is available to authorized users.

## Correspondence

Bipolar disorder (BD) has a lifetime prevalence of 4–5% in the general population. It is frequently associated with high rates of morbidity, mortality, and completed suicides (Mathers et al. 2006; Merikangas 2007; Nordentoft et al. 2011). It has been reported that only 20% of BD patients experiencing a depressive episode are diagnosed with BD within the first year of seeking treatment. This greatly underscores the need for objective diagnostic and vulnerability markers of this debilitating illness (Goldberg et al. 2001). Noticeably, previous epidemiological studies have reported that first-degree relatives of BD patients have an increased tenfold risk of BD as compared to the general population—which strongly highlights the role of genetic factors to the etiology of BD (Kessler et al. 1994; Olvet et al. 2013). However, despite these facts, there are no clinically useful biomarkers of vulnerability to BD that guides the institution of prophylactic interventions. These timely interventions may delay the onset of BD and translate into better clinical outcomes such as decreased rates of recurrence, less severe episodes (Post et al. 2010), and reduced medical related costs due to less hospitalizations.

Multiple studies have reported neurocognitive abnormalities in BD patients as compared to demographically matched healthy controls (HCs). These abnormalities have primarily been shown in key cognitive domains such as: executive function, sustained attention, verbal learning, and working memory (Robinson and Ferrier 2006; Torres et al. 2007; Arts et al. 2008; Bora et al. 2009; Torres et al. 2010; Mann-Wrobel et al. 2011; Bourne et al. 2013; Bauer et al. 2015; Wu et al. 2016). Furthermore, studies examining neurocognitive measurements in first-degree relatives of BD patients have also reported deficits in unaffected first-degree relatives in similar neurocognitive domains. A recent meta-analysis summarized studies investigating neurocognitive endophenotypes in BD and reported abnormalities in first-degree relatives of BD patients in key domains such as: set-shifting, processing speed, verbal learning, and response inhibition (Bora et al. 2009). Similarly, in a recent review, Olvet et al. reported a consistent theme on memory-related deficits in unaffected twins and siblings of patients with BD as compared to HCs (Olvet et al. 2013). Specifically, verbal, declarative, and working memory deficits were shown in unaffected siblings (Gourovitch et al. 1999; Kéri et al. 2001; Kieseppä et al. 2005; Christensen et al. 2006). Moreover, several other studies have highlighted executive function and verbal memory abnormalities as candidate endophenotypes of BD following reported deficits in these domains in first-degree relatives of BD patients (Arts et al. 2008; Bora et al. 2009; Doyle et al. 2009). However, while these studies have undeniably advanced our understanding of vulnerability markers of BD, it remains unknown whether reported abnormalities can objectively identify unaffected individuals vulnerable to BD and at an individual level. Noticeably, being able to predict an individual participant’s probability of vulnerability to BD based on a hazard-free and easily accessible neurocognitive task could help in institution of individualized prophylactic interventions and translate into favorable clinical outcomes.

**Fig. 1:**
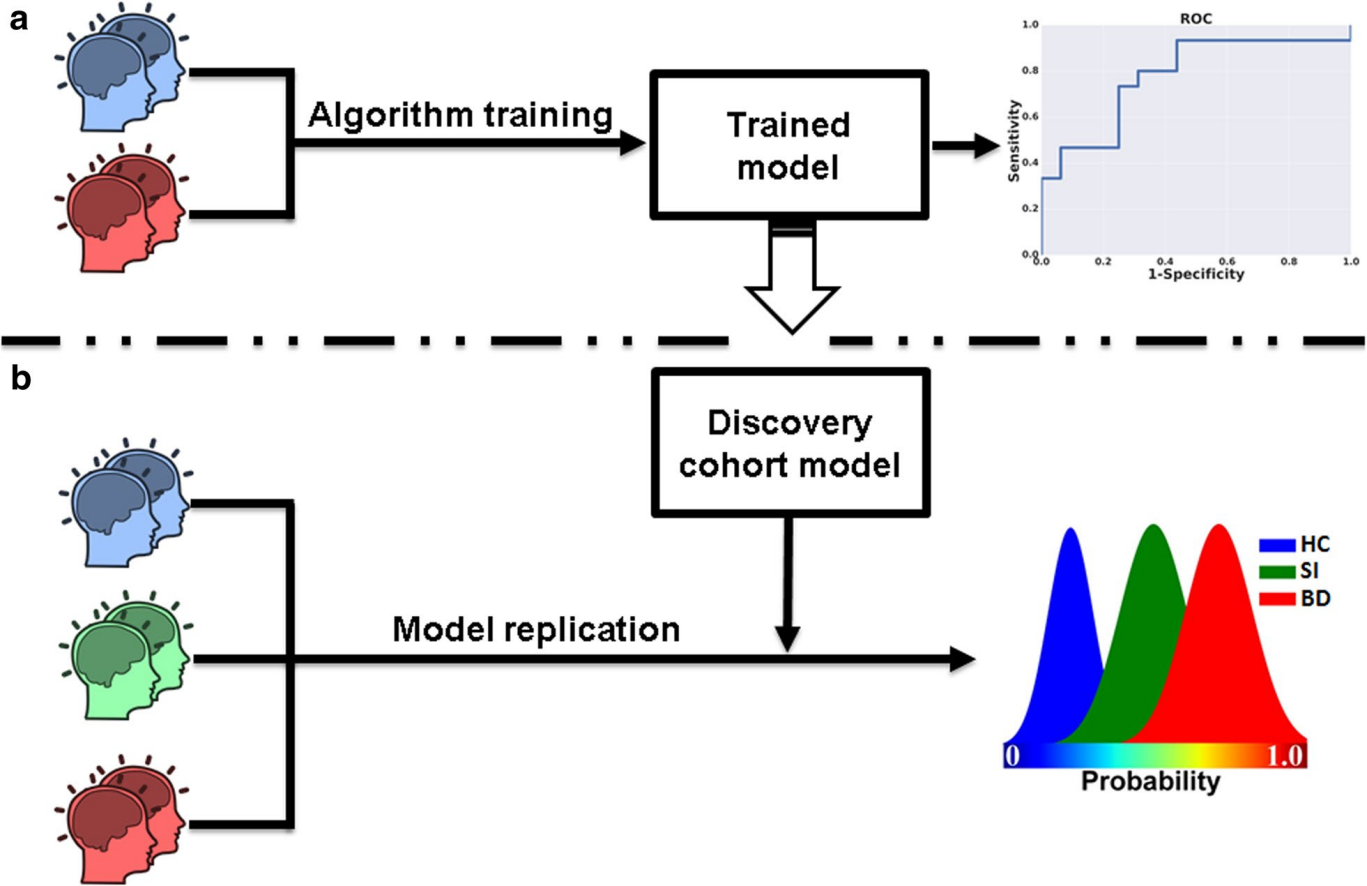
A flow diagram showing the signature discovery and replication stages

To achieve this objective, we recruited 21 euthymic BD patients (7 males, 14 females; age: 36.12 ± 16.55 years) and 21 demographically matched HCs (5 males, 16 females; age: 36.08 ± 12.66 years) at the University of North Carolina at Chapel Hill—a sample we refer to as the *discovery cohort*. A set of neurocognitive task scores were assessed for each individual using the Cambridge neuropsychological test automated battery (CANTAB). The nine assessed CANTAB neurocognitive tasks include: Affective Go/No-Go, Big/Little Circle, Cambridge Gambling Task, Choice Reaction Time, Motor Screening, Match to Sample Visual Search, Rapid Visual Processing, Spatial Recognition Memory, and Spatial Span task. The essence and measurements of all nine tasks are summarized in Table 1. As a second step, a *replication cohort* of 15 BD patients (5 males, 10 females; age: 32.67 ± 9.26 years) and 16 demographically matched HCs (5 males, 11 females; age: 33.75 ± 10.95 years) were assessed at the University of Texas Health Science Center at Houston. A set of CANTAB neurocognitive task measurements similar to the discovery cohort was also assessed. Notably, in the second center (replication cohort), an additional group of 15 age- and gender-matched siblings (SI) (4 males, 11 females; age: 32.20 ± 11.69 years) of BD patients (non-affected with BD) were also recruited and their CANTAB measurements were assessed. These data were first used to ‘train’ a least absolute shrinkage selection operator (LASSO) machine-learning algorithm in distinguishing patients from HCs. Second, the established predictive signature was further validated using an independent *replication cohort* of BD patients and HCs (Fig. 1). Lastly, the extent to which the validated predictive neurocognitive signature may differentiate the siblings (SIs) from HCs and BD patients was also examined.

**Table 1:** Cognitive tasks and measurements

No.	CANTAB task	Evaluation	Measurements
1	Affective Go/No-Go	Inhibition control	Reaction time*, accuracy
2	Big/Little Circle	Comprehension, learning and reversal	Reaction time*, accuracy
3	Cambridge Gambling Task	Risk-taking behavior	Reaction time*, accuracy, proportion bets across trials with more/equally/less likely outcome
4	Choice Reaction Time	Simple (motor) processing speed	Reaction time*, accuracy
5	Motor Screening	Simple (motor) processing speed	Reaction time*
6	Match to Sample Visual Search	Ability to match motor and visual stimuli	Reaction time*, accuracy
7	Rapid Visual Processing	Sustained attention	Reaction time*, accuracy
8	Spatial Recognition Memory	Visual spatial recognition memory	Reaction time*, accuracy
9	Spatial Span task	Spatial working memory	Span length, number of attempts, reaction times*

*Reaction time is in milliseconds

**Fig. 2:**
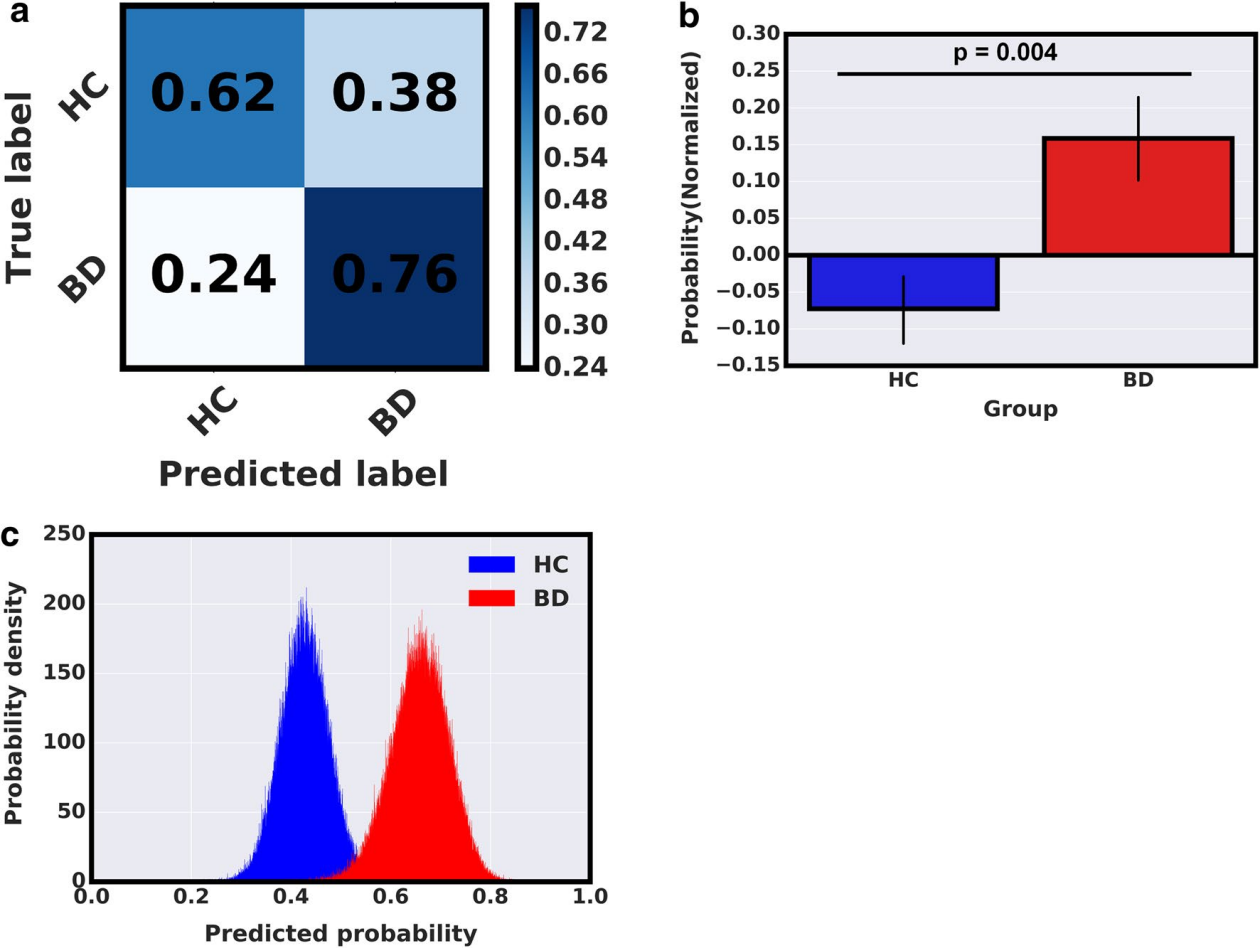
**a** A ‘confusion matrix’ depicting actual and LASSO predicted diagnostic labels in the discovery cohort.** b** A comparison of predicted probability scores between BD patients and HCs in the discovery cohort.** c** A bootstrapping calculation was performed to estimate distribution of the mean predicted probability for BD patients and HCs in the discovery cohort

**Fig. 3:**
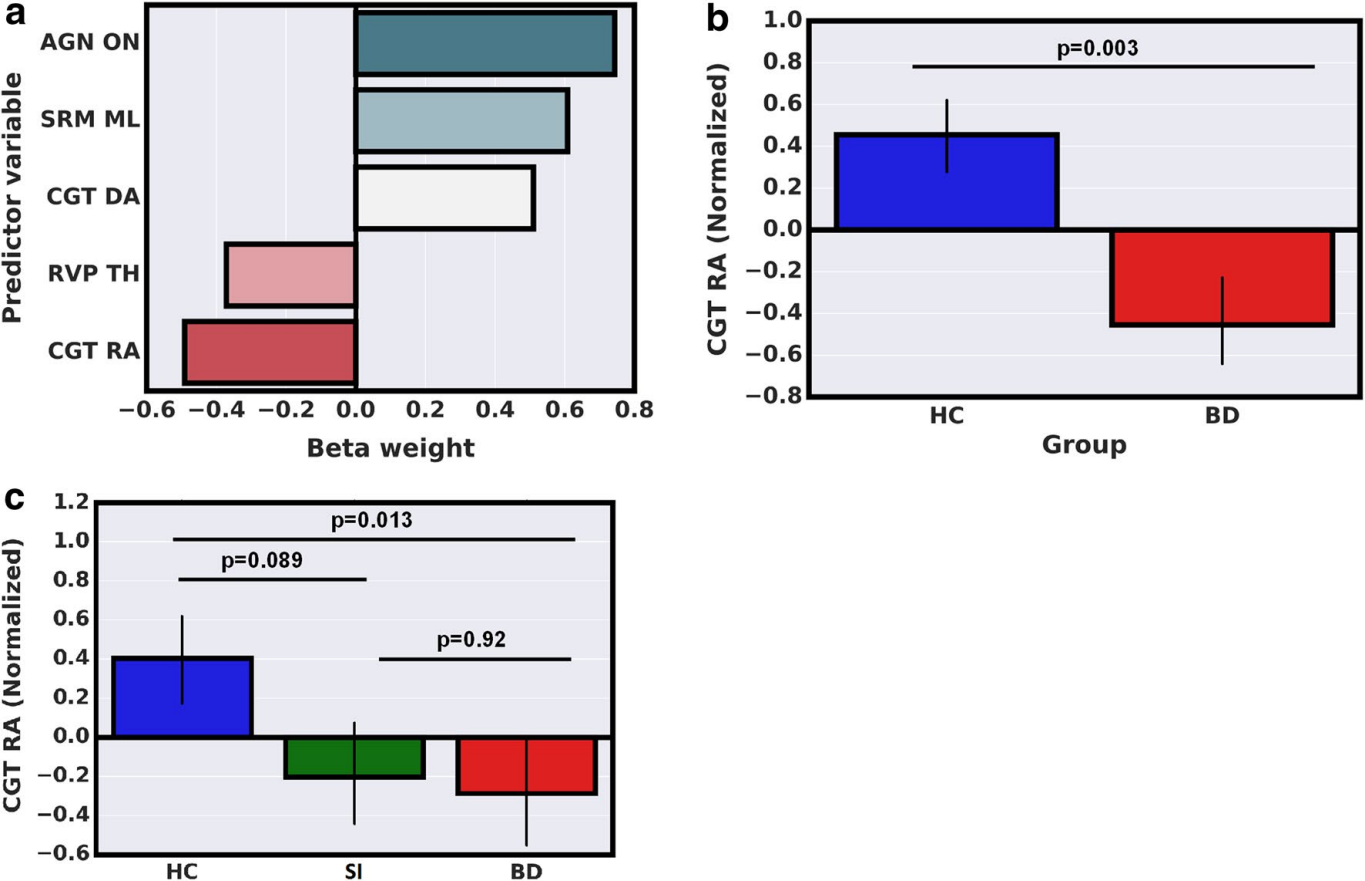
**a** A bar graph showing LASSO algorithm coefficients assigned to the most relevant CANTAB neurocognitive measurements.* AGN ON* affective* Go/No* go task total omission with negative stimuli,* SRM ML* spatial recognition memory task mean latency,* CGT DA* Cambridge gambling task delay aversion,* CGT RA* Cambridge gambling task risk adjustment,* RVP TH* rapid visual processing task total hits. These neurocognitive variables were assigned as non-zero coefficients during algorithm training. Positive coefficients represent increased neurocognitive scores in BD patients as compared to HCs and vice versa.** b** A bar graph comparing CGT RA scores from the discovery cohort.** c** A three-group (HCs, SIs, BD) comparison of CGT RA scores in the replication cohort

**Fig. 4:**
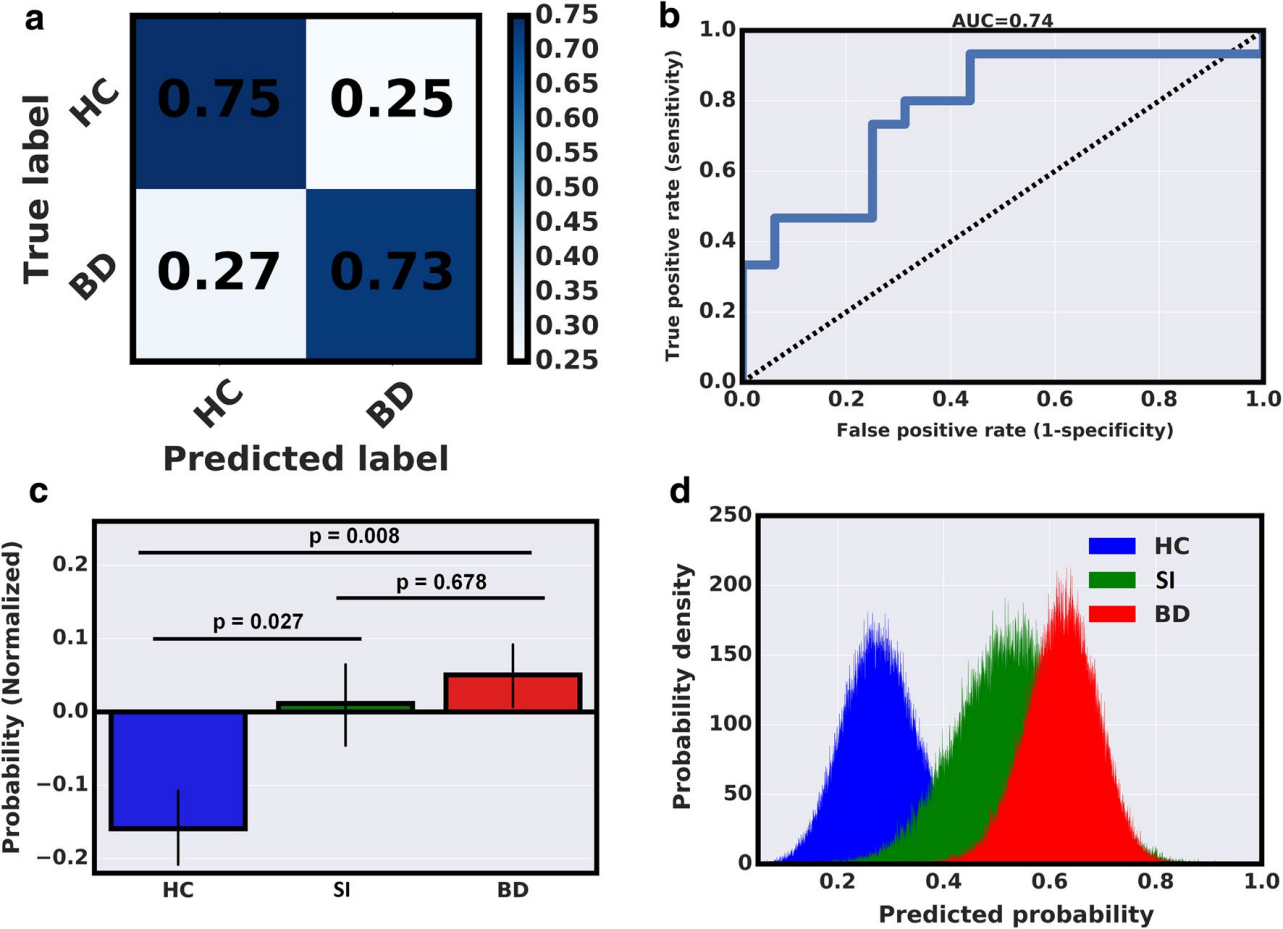
**a** A ‘confusion matrix’ representing actual and predicted patient and HCs labels in the replication cohort.** b** A receiver operating characteristic (ROC) curve depicting the algorithm’s performance in distinguishing BD patients from HCs in the replication cohort.** c** A bar graph comparing predicted probability scores between HCs, SIs, and BD patients HCs in the replication cohort.** d** A bootstrapping calculation was used to estimate the distribution of the mean predicted probability scores for BD patients, SIs, and HCs

The LASSO algorithm identified individual BD patients from HCs in the *discovery cohort* with 69% accuracy, 76% sensitivity, 62% specificity, 67% of positive predictive values (PPV), 72% of negative predictive values (NPV), and an area under receiver operating characteristic curve (AUROC) of 0.6905 with Chi-square *p* = 0.0126 (Fig. 2 and Additional file 1: Table S1). In the discovery cohort, predictor variables identified by the LASSO algorithm as most relevant in distinguishing BD patients from HCs (non-zero coefficients) include: number of omission errors to negative stimuli on the Affective Go/No-Go task, delay aversion, and the risk adjustment on the Cambridge Gambling Task and the total number of hits on the Rapid Visual Processing (Fig. 3 and Additional file 1: Table S2). In the replication cohort, the LASSO model derived at the discovery stage identified individual BD patients from HCs in the replication cohort with 74% accuracy, 73% sensitivity, 75% specificity, 73% of PPV, 75% of NPV, and an AUROC of 0.7417 (Fig. 4 and Additional file 1: Table S3). These predictions were significant (Chi-square *p* = 0.007). Predicted probability scores of HCs differed significantly from SIs and BD patients with *p* = 0.027 and *p* = 0.008, respectively. On the other hand, SIs were largely indistinguishable from BD patients with *p* = 0.678. These tests were performed using a non-parametric Kruskal–Wallis statistical test.

From a cognitive viewpoint, compared to HCs, individuals with BD committed a greater number of errors when exposed to negative stimuli. This finding provides further support for the presence of a negative affective bias which is reflected by impaired cognitive processing resulting from exposure to negative stimuli in both adults with BD and offspring of BD patients (Pavuluri and Passarotti 2008; Abe et al. 2011; Passarotti et al. 2011, 2012; Bauer et al. 2015). Furthermore, HCs had a higher quality of risk adjustment on the CGT task compared with individuals with BD (Quraishi and Frangou 2002), which is a reliable estimate of impulsivity and risk taking (Swann et al. 2003). Therefore, our findings are consistent with previous evidence that patients with BD have a high reward-seeking response and are unable to delay gratification (Najt et al. 2007; Swann et al. 2009). Moreover, in spite of the absence of a diagnosis of BD, the at-risk individuals displayed the tendency to make poorer decisions compared with HCs. This finding is particularly relevant because, to date, few studies have focused on the cognitive functioning of siblings of BD patients. Previous studies of unaffected siblings found that they scored lower on tests of verbal learning, attention, and planning than healthy individuals (Kéri et al. 2001; Trivedi et al. 2008; Kulkarni et al. 2010; Nehra et al. 2014). Further, in line with our findings, the magnitude of these cognitive deficits of SIs has consistently been reported to be intermediate between that of HCs and BD patients. Another potential implication of our findings is that impulsivity, a trait typically associated with BD (Newman and Meyer 2014) and underlying decision making and reward tasks (Christodoulou et al. 2006) is a potential marker of vulnerability to BD in SIs.

The current study has some potential limitations. The overall sample size in both discovery and replication cohorts were small and therefore our results should be regarded as preliminary. The discovery cohort was relatively small as we only considered euthymic patients at the signature discovery stage to avoid potential confounders related to mood phase (e.g., depression, mania). Six SI participants were diagnosed with other mood disorders other than BD (e.g., major depression) and future studies should examine this research question using an SI cohort without any psychiatric diagnoses. BD patients included in the discovery cohort were taking psychotropic medications which may be a potential confounder but also a reflection of standard clinical practice.

In conclusion, we report a study showing neurocognitive signature able to distinguish individual BD patients from HCs. We suggest this signature could be combined with other biological features to potentially develop a BD prediction model. However, the current study serves as a proof-of-concept. Future studies will examine this hypothesis using other biological markers (e.g., neuroimaging) as well as attempt to integrate multi-scale biomarkers (e.g., neuroimaging and neurocognition) which may potentially improve the current prediction results.

## Additional file

**Additional file 1.** Detailed prediction results in both discovery and replication cohorts.

## Abbreviations

BD: bipolar disorders
HC: healthy control
SI: sibling
CANTAB: Cambridge Neurocognitive Test Automated Battery
UNC: University of North Carolina at Chapel Hill
UTHealth: University of Texas Health Science Center at Houston
HAMD: Hamilton Depression Rating Scale
MADRS: Montgomery–Åsberg Depression Rating Scale
YMRS: Young Mania Rating Scale
LASSO: Least Absolute Selection Shrinkage Algorithm
PPV: positive predictive values
NPV: negative predictive values
AUROC: area under receiver operating characteristic curve
