# Erratum to: Prediction of vulnerability to bipolar disorder using multivariate neurocognitive patterns: a pilot study

**DOI:** 10.1186/s40345-017-0105-5

**Published:** 2017-09-18

**Authors:** Mon-Ju Wu, Benson Mwangi, Ives Cavalcante Passos, Isabelle E. Bauer, Bo Cao, Thomas W. Frazier, Giovana B. Zunta-Soares, Jair C. Soares

**Affiliations:** 1 UT Center of Excellence on Mood Disorder, Department of Psychiatry and Behavioral Sciences, The University of Texas Science Center at Houston, Houston, TX USA; 2 Cleveland Clinic Children’s Hospital Center for Pediatric Behavioral Health, Cleveland, OH USA; 3 Department of Psychiatry & Behavioral Sciences, The University of Texas Health Science Center, 1941 East Road, Houston, TX 77054 USA

## Erratum to: Int J Bipolar Disord (2017) 5:32 DOI 10.1186/s40345-017-0101-9

In the original version of this article (Wu et al. 2017), published on 1 September 2017, the name of author ‘Bo Cao’ was wrongly displayed. In this Erratum the incorrect name and correct name are shown. The original publication of this article has been corrected.

Originally the author name has been published as:

- Cao Bo

The correct author name is:

- Bo Cao
