# Supplementary material for: Prediction of vulnerability to bipolar disorder using multivariate neurocognitive patterns: a pilot study
Source: Int J Bipolar Disord. 2017 Sep 1;5:32. doi: 10.1186/s40345-017-0101-9 (PMC5578943; doi:10.1186/s40345-017-0101-9)
Supplement: Supplementary file 1 — Additional file 1. Detailed prediction results in both discovery and replication cohorts. [file 40345_2017_101_MOESM1_ESM.docx]

**Additional File: Detailed prediction results in both discovery and replication cohorts**

**Table S1. Discovery cohort demographics details**

| **Subjects  (Con- Control)**  **(AR- At risk)**  **(Pat- Patient)** | **Age** | **Gender** | **Subject Diagnosis** | **Model**  **Prediction** | **Probability** | **Medication Status**  **(0-Medication naïve)**  **(1-Medicated)** |
| --- | --- | --- | --- | --- | --- | --- |
| Con01 | 20.00 | Female | Healthy | Healthy | 0.3641 | 0 |
| Con02 | 24.32 | Female | Healthy | Healthy | 0.3410 | 0 |
| Con03 | 23.93 | Female | Healthy | Healthy | 0.0355 | 0 |
| Con04 | 20.00 | Female | Healthy | Healthy | 0.2194 | 0 |
| Con05 | 19.88 | Male | Healthy | Healthy | 0.2471 | 0 |
| Con06 | 57.61 | Male | Healthy | Bipolar | 0.5258 | 0 |
| Con07 | 21.00 | Female | Healthy | Bipolar | 0.5253 | 0 |
| Con08 | 28.93 | Female | Healthy | Healthy | 0.4960 | 0 |
| Con09 | 54.02 | Male | Healthy | Healthy | 0.2846 | 0 |
| Con10 | 26.70 | Male | Healthy | Healthy | 0.2729 | 0 |
| Con11 | 55.45 | Female | Healthy | Bipolar | 0.5791 | 0 |
| Con12 | 44.00 | Male | Healthy | Healthy | 0.4720 | 0 |
| Con13 | 55.79 | Female | Healthy | Bipolar | 0.6216 | 0 |
| Con14 | 48.60 | Female | Healthy | Bipolar | 0.6616 | 0 |
| Con15 | 22.36 | Female | Healthy | Bipolar | 0.6215 | 0 |
| Con16 | 22.00 | Female | Healthy | Healthy | 0.3964 | 0 |
| Con17 | 51.84 | Female | Healthy | Bipolar | 0.5952 | 0 |
| Con18 | 20.64 | Female | Healthy | Healthy | 0.4463 | 0 |
| Con19 | 55.35 | Male | Healthy | Bipolar | 0.9644 | 0 |
| Con20 | 64.65 | Male | Healthy | Healthy | 0.0163 | 0 |
| Con21 | 21.43 | Female | Healthy | Healthy | 0.2865 | 0 |
| Pat01 | 32.03 | Female | Bipolar | Healthy | 0.3325 | 1 (Psychiatric meds, anticonvulsants, mood stabilizers) |
| Pat02 | 29.08 | Female | Bipolar | Bipolar | 0.7750 | 1 (Psychiatric meds, benzodiazepines, antipsychotics, mood stabilizers) |
| Pat03 | 46.88 | Female | Bipolar | Bipolar | 0.7073 | 1 (Psychiatric meds) |
| Pat04 | 28.76 | Male | Bipolar | Healthy | 0.1297 | 0 |
| Pat05 | 50.60 | Female | Bipolar | Bipolar | 0.6673 | 1 (Psychiatric meds) |
| Pat06 | 19.19 | Female | Bipolar | Bipolar | 0.6784 | 1 (Psychiatric meds, mood stabilizers) |
| Pat07 | 22.66 | Female | Bipolar | Bipolar | 0.8546 | 1 (Psychiatric meds, anticonvulsants, antidepressants, atypical antipsychotics, benzodiazepines, antipsychotics, mood stabilizers) |
| Pat08 | 55.13 | Female | Bipolar | Bipolar | 0.8903 | 1 (Psychiatric meds, mood stabilizers) |
| Pat09 | 33.00 | Female | Bipolar | Healthy | 0.1392 | 1 (Psychiatric meds, antipsychotics, mood stabilizers) |
| Pat10 | 38.00 | Female | Bipolar | Bipolar | 0.9106 | 1 (Psychiatric meds, antipsychotics, mood stabilizers) |
| Pat11 | 22.99 | Male | Bipolar | Bipolar | 0.7956 | 1 (Psychiatric meds, anticonvulsants, atypical antipsychotics, mood stabilizers, benzodiazepines, stimulants, antipsychotics) |
| Pat12 | 56.60 | Female | Bipolar | Bipolar | 0.6198 | 1 (Psychiatric meds, mood stabilizers) |
| Pat13 | 41.95 | Male | Bipolar | Healthy | 0.1976 | 1 (Psychiatric meds, lithium, anticonvulsants, atypical antipsychotics, mood stabilizers, antidepressants) |
| Pat14 | 32.63 | Female | Bipolar | Bipolar | 0.7502 | 0 |
| Pat15 | 54.98 | Female | Bipolar | Bipolar | 0.8251 | 1 (Psychiatric meds, lithium, antidepressants, antipsychotics, mood stabilizers) |
| Pat16 | 43.39 | Female | Bipolar | Bipolar | 0.8339 | 1 (Psychiatric meds, benzodiazepines, mood stabilizers, antipsychotics) |
| Pat17 | 19.43 | Female | Bipolar | Bipolar | 0.8577 | 1 (Psychiatric meds, atypical antipsychotics, mood stabilizers, antipsychotics, lithium) |
| Pat18 | 20.36 | Male | Bipolar | Bipolar | 0.5201 | 1 (Psychiatric meds, stimulants) |
| Pat19 | 48.12 | Female | Bipolar | Bipolar | 0.9580 | 1 (Psychiatric meds, anticonvulsants, mood stabilizers, benzodiazepines) |
| Pat20 | 22.44 | Female | Bipolar | Bipolar | 0.9479 | 1 (Psychiatric meds) |
| Pat21 | 39.40 | Male | Bipolar | Healthy | 0.4401 | 1 (Psychiatric meds, mood stabilizers, antipsychotics, stimulants) |

**Table S2. Neurocognitive variables and assigned coefficients**

| **Task** | **Measurement** | **Beta weights** | **Beta percentage** |
| --- | --- | --- | --- |
| AGN | Total omissions negative stimuli | 0.743 | 22.00% |
| SRM | Mean latency | 0.6079 | 18.00% |
| CGT | Delay aversion | 0.5099 | 15.10% |
| CGT | Risk adjustment | -0.4911 | 14.54% |
| RVP | Total hits | -0.3711 | 10.99% |
| CGT | Risk taking | -0.2439 | 7.22% |
| CGT | Overall proportion bet | -0.1716 | 5.08% |
| CRT | Mean latency | 0.0935 | 2.77% |
| CRT | Percent correct trials | -0.052 | 1.54% |
| AGN | Mean correct latency positive | 0.0328 | 0.97% |
| AGN | Total commissions negative | 0.0259 | 0.77% |
| AGN | Mean correct latency negative | 0.0144 | 0.43% |
| SRM | Number correct | -0.0073 | 0.22% |
| BLC | Percent correct | -0.0062 | 0.18% |
| MTS | Percent correct | -0.006 | 0.18% |
| AGN | Total omissions neutral | 0.0007 | 0.02% |
| AGN | Total commissions positive | 0 | 0.00% |
| AGN | Total commissions neutral | 0 | 0.00% |
| AGN | Total omissions positive | 0 | 0.00% |
| AGN | Total omissions negative | 0 | 0.00% |
| AGN | Total omissions positive stimuli | 0 | 0.00% |
| BLC | Mean correct latency | 0 | 0.00% |
| BLC | Total errors | 0 | 0.00% |
| CGT | Deliberation time | 0 | 0.00% |
| CGT | Quality of decision making | 0 | 0.00% |
| CRT | Percent commission trials | 0 | 0.00% |
| MOT | Mean latency | 0 | 0.00% |
| MTS | Total correct | 0 | 0.00% |
| RVP | Total correct rejections | 0 | 0.00% |
| SRM | Mean correct latency | 0 | 0.00% |
| SRM | Number incorrect | 0 | 0.00% |
| SRM | Percent correct | 0 | 0.00% |
| SRM | Percent incorrect | 0 | 0.00% |
| SSP | Mean time to first response/#attempts | 0 | 0.00% |
| SSP | Mean time to last response/#attempts | 0 | 0.00% |
| SSP | Span length | 0 | 0.00% |
| SSP | Total errors | 0 | 0.00% |

**Table S3. Replication cohort demographics details**

| **Subjects** **(Con- Control)**  **(SI- Sibling)**  **(Pat- Patient)** | **Age** | **Gender** | **Subject Diagnosis** | **Model**  **Prediction** | **Probability** | **Medication Status**  **(0-Medication naïve)**  **(1-Medicated)** |
| --- | --- | --- | --- | --- | --- | --- |
| Con01 | 23 | Male | Healthy | Healthy | 0.2018 | 0 |
| Con02 | 48 | Male | Healthy | Healthy | 0.4537 | 0 |
| Con03 | 33 | Female | Healthy | Bipolar | 0.6220 | 0 |
| Con04 | 20 | Female | Healthy | Bipolar | 0.5900 | 0 |
| Con05 | 22 | Female | Healthy | Healthy | 0.1411 | 0 |
| Con06 | 25 | Female | Healthy | Bipolar | 0.6147 | 0 |
| Con07 | 51 | Female | Healthy | Healthy | 0.1511 | 0 |
| Con08 | 43 | Female | Healthy | Healthy | 0.2090 | 0 |
| Con09 | 26 | Male | Healthy | Bipolar | 0.6341 | 0 |
| Con10 | 48 | Female | Healthy | Healthy | 0.4415 | 0 |
| Con11 | 34 | Female | Healthy | Healthy | 0.0887 | 0 |
| Con12 | 36 | Female | Healthy | Healthy | 0.1723 | 0 |
| Con13 | 25 | Male | Healthy | Healthy | 0.2957 | 0 |
| Con14 | 31 | Female | Healthy | Healthy | 0.4927 | 0 |
| Con15 | 50 | Male | Healthy | Healthy | 0.1634 | 0 |
| Con16 | 25 | Female | Healthy | Healthy | 0.1784 | 0 |
| SI01 | 52 | Male | Sibling^*^ | Healthy | 0.4320 | 0 |
| SI02 | 43 | Female | Sibling^*^ | Bipolar | 0.8436 | 0 |
| SI03 | 30 | Male | Sibling^*^ | Healthy | 0.4452 | 0 |
| SI04 | 29 | Male | Sibling^*^ | Healthy | 0.0424 | 0 |
| SI05 | 19 | Female | Sibling^**^ | Bipolar | 0.6459 | 1 (Stimulants, psychiatric meds) |
| SI06 | 55 | Male | Sibling^*^ | Healthy | 0.4580 | 0 |
| SI07 | 24 | Female | Sibling^*^ | Healthy | 0.2814 | 1 (Psychiatric meds) |
| SI08 | 24 | Female | Sibling^**^ | Healthy | 0.3753 | 1 (Psychiatric meds) |
| SI09 | 23 | Female | Sibling^**^ | Bipolar | 0.7702 | 0 |
| SI10 | 24 | Female | Sibling^*^ | Healthy | 0.1936 | 0 |
| SI11 | 50 | Female | Sibling^**^ | Bipolar | 0.6177 | 0 |
| SI12 | 29 | Female | Sibling^*^ | Bipolar | 0.5779 | 0 |
| SI13 | 28 | Female | Sibling^*^ | Bipolar | 0.6603 | 0 |
| SI14 | 26 | Female | Sibling^**^ | Bipolar | 0.6560 | 1 (Psychiatric meds) |
| SI15 | 27 | Female | Sibling^**^ | Bipolar | 0.6736 | 1 (Psychiatric meds) |
| Pat01 | 21 | Male | Bipolar | Bipolar | 0.5517 | 1 (Psychiatric meds, antipsychotics, mood stabilizers, anticonvulsants) |
| Pat02 | 45 | Female | Bipolar | Bipolar | 0.5372 | 1 (Psychiatric meds, antipsychotics, mood stabilizers, atypical antipsychotics, mood stabilizers, antidepressants) |
| Pat03 | 40 | Female | Bipolar | Bipolar | 0.7018 | 1 (Psychiatric meds, mood stabilizers, antipsychotics, benzodiazepines, atypical antipsychotics, antidepressants) |
| Pat04 | 26 | Female | Bipolar | Healthy | 0.4251 | 1 (Psychiatric meds) |
| Pat05 | 45 | Male | Bipolar | Bipolar | 0.7083 | 1 (Psychiatric meds, mood stabilizers, antipsychotics, atypical antipsychotics) |
| Pat06 | 34 | Female | Bipolar | Bipolar | 0.6299 | 1 (Psychiatric meds, mood stabilizers) |
| Pat07 | 31 | Male | Bipolar | Bipolar | 0.5025 | 1 (Psychiatric meds, antipsychotics, atypical antipsychotics, antidepressants) |
| Pat08 | 28 | Male | Bipolar | Healthy | 0.0860 | 1 (Psychiatric meds, antipsychotics, mood stabilizers, atypical antipsychotics) |
| Pat09 | 51 | Male | Bipolar | Bipolar | 0.5057 | 1 (Psychiatric meds, antipsychotics, mood stabilizers) |
| Pat10 | 20 | Female | Bipolar | Bipolar | 0.6505 | 1 (Psychiatric meds, mood stabilizers, antipsychotics, atypical antipsychotics, antidepressants) |
| Pat11 | 25 | Female | Bipolar | Bipolar | 0.6553 | 1 (Psychiatric meds, antipsychotics, mood stabilizers) |
| Pat12 | 35 | Female | Bipolar | Bipolar | 0.7809 | 1 (Psychiatric meds, mood stabilizers, antipsychotics, atypical antipsychotics, antidepressants, anticonvulsants) |
| Pat13 | 33 | Female | Bipolar | Bipolar | 0.6247 | 1 (Psychiatric meds, mood stabilizers, antipsychotics, atypical antipsychotics, antidepressants, stimulants) |
| Pat14 | 24 | Female | Bipolar | Healthy | 0.4135 | 1 (Psychiatric meds, antipsychotics, atypical antipsychotics, mood stabilizers, benzodiazepines, antidepressants, anticonvulsants) |
| Pat15 | 32 | Female | Bipolar | Healthy | 0.4834 | 1 (Psychiatric meds, antipsychotics, mood stabilizers) |

Sibling^*^- Sibling of BD patients who has no diagnosis; Sibling^**^- Sibling of BD patients with diagnosis of MDD or alcohol abuse but not diagnosed as BD nor schizophrenia.
